# Brain Death Knowledge and Attitudes Among Fourth-Year Medical Students at Umm Al-Qura University

**DOI:** 10.7759/cureus.69247

**Published:** 2024-09-12

**Authors:** Omar Basubrain

**Affiliations:** 1 Faculty of Medicine, Department of Medicine, Umm Al-Qura University, Makkah, SAU

**Keywords:** knowledge, attitude, saudi arabia, life support, medical students, organ donation, brain death

## Abstract

Background

Despite clear definitions of brain death, students and medical professionals may have varying understandings. This study investigated the knowledge and attitudes of medical students regarding brain death.

Methods

A cross-sectional survey was administered to 142 fourth-year medical students at Umm Al-Qura University. The survey used single-choice and Likert scale questions to assess knowledge of brain death definition, diagnosis procedures, and student attitudes. Prior to administration, the questionnaire underwent content validation by experts in medicine, ethics, and public health.

Results

A significant proportion of students lacked knowledge of the legal definition of brain death in Saudi Arabia (26.1%), brain death diagnostic procedures (43.7%), and prerequisites for declaring brain death (38%). While some students expressed confidence in diagnosing brain death, concerns about misdiagnosis were also present.

Conclusion

Fourth-year medical students in this study demonstrated significant knowledge gaps regarding brain death. Medical education programs may need to be revised to provide more comprehensive training on brain death and its implications. Additionally, public awareness campaigns could improve understanding and facilitate informed decision-making about organ donation. Further research, including multicenter studies, is warranted to confirm these findings and guide educational interventions.

## Introduction

Despite well-defined legal and medical definitions of brain death, knowledge gaps persist among healthcare professionals and the public [1]. Furthermore, the attitudes of medical staff and patient families significantly impact the donation process [2]. Accurate diagnosis is essential in transplantation [1], but public awareness of brain death and organ donation remains limited [3]. Studies also show insufficient knowledge of brain death criteria among various healthcare professionals [4-6]. These gaps can diverge from established guidelines [7,8].

Brain death definitions have become more specific since the initial 1968 Harvard criteria of "irreversible cessation of whole-brain activity" [9]. The Uniform Determination of Death Act (UDDA) in 1981 established two criteria, irreversible circulatory/respiratory cessation or complete brain function cessation (including brainstem death), neither of which can be reversed [10]. While not all brain cells die immediately, the World Brain Death Project offers standardized protocols requiring 100% certainty in clinical exams and auxiliary tools for brain death declaration [11]. These tools have 100% sensitivity and specificity for brain death identification [12].

However, low deceased donor transplant rates persist [13]. Potential reasons include the religious beliefs of families, chaplain involvement, and healthcare professionals' understanding of brain death [13-15]. Notably, research suggests doctors' perspectives on death may evolve throughout their medical careers, potentially starting in preclinical years [15].

Studies reveal a concerning knowledge gap in medical students' understanding of brain death [8,16-19]. For instance, a US study found only 33% of students grasped the concept fully [18]. Similarly, large studies in Spain and Mexico reported that just 67% and 39% of medical students, respectively, demonstrated adequate knowledge [18,19]. Notably, a Canadian study found no improvement in understanding organ donation over time, suggesting this knowledge gap may persist even after graduation [8].

Studies show international variations in brain death understanding [20-22]. While Iranian and Polish students largely viewed brain death as irreversible and supported organ donation after education [20,21], a concerning 40% of Saudi students believed it to be reversible [21]. However, a Canadian study found a more nuanced understanding, with 76% grasping the concept of brain death despite a heartbeat [22]. Western Europe exhibits the highest organ donation rates, and research suggests a link between cultural perspectives on brain death and these donation rates [18].

Prior research suggests medical students' knowledge of brain death can improve after graduation [18,23]. This finding is particularly relevant considering the current curriculum in China, which lacks a dedicated brain death course. Although related topics are integrated into various courses, such as medical ethics in the third year and diagnosis covered in later electives like critical care medicine and surgery [24], this curricular structure may provide some exposure to brain death, but its brevity raises concerns about equipping students to identify donors and diagnose effectively.

The knowledge gap extends beyond students. While specialists like neurosurgeons routinely encounter brain death, diagnosis often falls to other specialists in various settings like general surgery or pediatrics [25]. Furthermore, clinicians across specialties engage in end-of-life discussions and advise on organ donation, requiring a strong understanding of brain death [25]. However, achieving consensus among professionals on the definition, irreversibility, and distinction from a persistent vegetative state appears challenging [26,27]. This, combined with potential medical school curriculum shortcomings [22], raises concerns about healthcare professionals' preparedness to manage brain death and support families.

Physicians play a key role in shaping public views on brain death and organ donation by educating patients and families [28]. While calls to integrate brain death curricula into medical education are frequent [17,28,29], current approaches may not adequately address the core concept of "brain death" itself. Research on medical professionals' understanding of organ donation exists [21,22,28], but studies specifically focused on medical students' comprehension of brain death are limited [17,21,28,29]. Additionally, these studies come from diverse countries with potentially varying curricula, limiting generalizability and hindering efforts to advocate for improved undergraduate education in brain death identification.

## Materials and methods

Study design and participants

This study employed a cross-sectional design to investigate medical students' knowledge and attitudes regarding brain death. The study was conducted at Umm Al-Qura University over a period of four weeks. Ethical approval was obtained from the Umm Al-Qura University Biomedical Research Ethics Committee (approval number: HAPO-02-K-012-2024-06-2182). The sample size was calculated using a 95% confidence interval and a 5% margin of error, resulting in a minimum sample size of 142. Researchers distributed a self-administered survey to fourth-year medical students. The online questionnaire was accessible for a predetermined period of two weeks.

The study included only fourth-year medical students enrolled at Umm Al-Qura University, regardless of gender. Students from other disciplines, students in lower academic years, and those who declined participation were excluded. A total of 142 medical students completed the survey, providing a response rate of 59%, based on the year class size of 240 students.

Survey development and validation

The researchers developed a questionnaire consisting of two sections. The first section assessed knowledge of brain death concepts and evaluation procedures through single-choice questions. The second section evaluated attitudes and beliefs related to brain death using a combination of multiple-choice and Likert scale questions.

To gauge students' existing knowledge of brain death, a 5-point Likert scale was employed, where 1 signified "no knowledge" and 5 represented "expert knowledge." This allowed for a nuanced assessment of their familiarity with the topic.

Prior to administration, the questionnaire underwent content validation by a panel of five experts consisting of two physicians specializing in neurology and critical care, a medical ethicist, and two public health researchers. The experts evaluated each item for clarity, relevance to brain death knowledge and attitudes, and comprehensiveness in covering key aspects of the topic. The content validity index (CVI) was calculated for each item, and items with a CVI below 0.8 were revised or removed based on expert feedback. The final questionnaire demonstrated a high degree of content validity. A copy of the questionnaire is provided in the Appendices.

Informed consent and data collection

Informed consent was obtained from all participants electronically before they could access the online survey. The consent form detailed the study's purpose, procedures, potential risks, and benefits and emphasized voluntary participation and the right to withdraw at any time.

The online survey was distributed via WhatsApp groups to all eligible fourth-year medical students. Reminder messages were sent three times a week to encourage participation. Data collection was conducted through the online survey platform, and responses were automatically stored in a secure database.

Data analysis

Data analysis was performed using Microsoft Excel for descriptive statistics, presenting frequencies and percentages for categorical variables. For ordinal data collected through Likert scales, appropriate non-parametric statistical tests were employed to examine potential differences in responses between students. The level of statistical significance was set at p<0.05.

## Results

A total of 142 medical students completed a survey regarding their understanding of brain death. The participants consisted of 66 females and 76 males. Among the 142 students, 85 had taken a course in neurology, while 57 had not.

Upon querying the students about their existing knowledge of brain death and grading them on a scale of 1-5, with 1 signifying no knowledge and 5 indicating expert knowledge, approximately 26% of the students indicated that they were devoid of any knowledge regarding the legal definition of brain death in Saudi Arabia. Furthermore, just 5.6% of the participants claimed to possess expertise in this domain. Additionally, only five students demonstrated expert-level knowledge pertaining to the presence of a policy on testing and announcing brain death. On the other hand, 29% of the participants were unaware of any such policy. The results obtained for the students' knowledge of the apnea test procedure and its interpretation, brainstem reflexes to assess during a brain death examination, and the prerequisites necessary before conducting a brain death examination were comparable, implying that only a few students possessed expert-level knowledge of these parameters. The findings have been summarized in Table 1.

**Table 1:** Awareness of medical students regarding brain death on a scale of 1-5 (1=no knowledge to 5=expert)

	Knowledge level (1 being no knowledge to 5 being expert)	Frequency (n=142)	Percentage (%)
Legal definition of brain death in Saudi Arabia	1	37	26.1
	2	28	19.7
	3	53	37.3
	4	16	11.3
	5	8	5.6
Presence of a policy on testing and announcing brain death	1	41	28.9
	2	41	28.9
	3	33	23.2
	4	22	15.5
	5	5	3.5
The apnea test procedure and its interpretation	1	62	43.7
	2	34	23.9
	3	30	21.1
	4	11	7.7
	5	5	3.5
Brainstem reflexes to assess during a brain death examination	1	54	38
	2	36	25.4
	3	32	22.5
	4	14	9.9
	5	6	4.2
The prerequisites necessary before conducting a brain death examination	1	54	38
	2	36	25.4
	3	32	22.5
	4	14	9.9
	5	6	4.2

The findings from the study revealed that 21.8% of the participants had no knowledge of the current diagnostic criteria for brain death, while 16.9% believed the criteria to be irreversible loss of brainstem function. A substantial proportion of students (28.9%) believed that the criteria were irreversible loss of function of all brain cells and irreversible loss of function of both hemispheres and the midbrain, and 14% believed it to be irreversible loss of function of the cerebral cortex.

In response to questions about the symptoms and conditions of brain-dead individuals, the majority of participants (67%) replied no when asked if patients with brain death experience pain, while 19% replied yes, and 33% were unsure. Similarly, when asked if brain-dead patients could awaken or regain their neurological function, the majority (67%) replied no, while some said yes, and many were unsure. The exact numbers and percentages of respondents are provided in Table 2.

**Table 2:** Questions about symptoms and conditions in brain-dead individual

Question	Answer choices	Frequency (n=142)	Percentage %
Do brain-dead patients feel pain?	I do not know	38	26.8
	No	77	54.2
	Yes	27	19
Can brain-dead patients wake up or regain neurological function?	I do not know	31	21.8
	No	104	73.2
	Yes	7	4.9
Which of the following symptoms excludes brain death?	All of the above	88	62
	Flexion movements of the fingers of the hand	14	9.9
	Present pupillary reaction to light	22	15.5
	Preservation of tendon reflexes	14	9.9
	Subtle periodic and rhythmic contractions of facial muscles	4	2.8
Is it always necessary to perform an instrumental examination in the procedure for pronouncing brain death?	Depending on the case	47	33.1
	I do not know	53	37.3
	No	10	7
	Yes	32	22.5
Is it possible to maintain pregnancy in women diagnosed with brain death on artificial life support?	I do not know	85	59.9
	No, because the brain stem has died	13	9.2
	Yes, because the brain structures outside the brainstem are responsible for maintaining the pregnancy	44	31
Do brain death, a persistent vegetative state, and locked-in syndrome represent the same medical conditions?	I do not know	82	57.7
	No	41	28.9
	Yes	19	13.4
Is there a fatwa regarding brain death in Saudi Arabia?	I do not know	86	60.6
	No	7	4.9
	Yes	49	34.5

The participants were presented with a selection of symptoms to choose from to exclude brain death, including (1) flexion movements of the fingers of the hand, (2) pupillary reaction to light, (3) preservation of tendon reflexes, and (4) subtle periodic and rhythmic contractions of the facial muscles. The majority of students (66%) replied that all of the above were symptoms that could exclude brain death.

The questions posed in the survey covered topics such as the necessity of conducting an instrumental examination in determining brain death, the feasibility of maintaining a pregnancy in women diagnosed with brain death and receiving artificial life support, and the distinction between brain death, persistent vegetative state, and locked-in syndrome. Additionally, the survey included a query about the existence of a fatwa regarding brain death in Saudi Arabia. Responses to these inquiries are summarized in Table 2.

The survey assessed participants' level of agreement with a series of statements on a scale of 1-5, with 1 representing strong disagreement and 5 representing strong agreement. The statements included the following: (1) agreement with the diagnosis of brain death, (2) confidence in explaining the concept of brain death to patients and their families, (3) the importance of organ donation after brain death as an option for families, (4) the challenges posed by cultural or religious beliefs in discussing brain death with families, (5) the possibility of being misdiagnosed as brain dead and missing out on treatment, (6) the possibility of being misdiagnosed as brain dead and having organs and/or tissues harvested while still alive, and (7) the potential for premature termination of treatment if organ donation after brain death is pursued.

The results indicated a mixed pattern of agreement and confidence in brain death diagnoses. While medical students generally believed that misdiagnosis was possible and that organ donation could occur while the patient was still alive, the findings are presented in Table 3.

**Table 3:** Attitudes of medical students towards brain death and organ harvest (on a scale of 1-5 (1=strongly disagree to 5=strongly agree))

Question	Responses	Frequency (n=142)	Percentage
I am in agreement with brain death diagnosis	1	19	13.4
	2	14	9.9
	3	57	40.1
	4	28	19.7
	5	24	16.9
I feel confident in my ability to explain the concept of brain death to patients and their families	1	36	25.4
	2	44	31
	3	45	31.7
	4	10	7
	5	7	4.9
I believe that organ donation after brain death is an important option for families to consider	1	19	13.4
	2	17	12
	3	41	28.9
	4	30	21.1
	5	35	24.6
Cultural or religious beliefs sometimes make it difficult to discuss brain death with families	1	17	12
	2	18	12.7
	3	41	28.9
	4	23	16.2
	5	43	30.3
I might be misdiagnosed as brain dead and lose the chance of treatment	1	25	17.6
	2	38	26.8
	3	55	38.7
	4	14	9.9
	5	10	7
I might be misdiagnosed as brain dead and my organs and/or tissues might be harvested while I am still alive	1	23	16.2
	2	39	27.5
	3	49	34.5
	4	21	14.8
	5	10	7
My treatment may be terminated prematurely if I declared to donate organs after brain death	1	33	23.2
	2	29	20.4
	3	59	41.5
	4	13	9.2
	5	8	5.6

## Discussion

Knowledge gaps in brain death recognition

Despite a well-established legal definition of brain death, public and medical professional understanding remains varied [1]. Accurate brain death diagnosis is crucial for ethical organ transplantation [1]. However, studies suggest a knowledge gap among physicians, particularly those outside critical care specialties, regarding the latest brain death criteria [2-4]. This study aimed to assess medical students' knowledge and attitudes towards brain death and organ donation in their fourth year of medical school.

The survey results revealed concerning knowledge gaps among students, even in their later years of medical education. A significant portion lacked understanding of the core concepts of brain death, including the legal definition in Saudi Arabia, apnea testing procedures, brainstem reflex assessment, and prerequisites for declaration. This may be attributed to incomplete neurology coursework. These findings align with previous research on medical students' knowledge of brain death [16,22].

The importance of differentiating brain death from other conditions

Accurate differentiation between coma, persistent vegetative state, and brain death is critical. Brain death signifies legal death, characterized by irreversible coma with absent responsiveness, brainstem reflexes, and respiratory function. Additionally, a diagnosis requires clinical or neuroimaging evidence of acute brain injury consistent with permanent neurological loss [29]. Similar to the current study, research by Shrivastav et al. [30] identified a concerning lack of awareness regarding brain death criteria among healthcare professionals.

Knowledge improvement and educational needs

While the current study observed a higher proportion of students with accurate answers compared to Tawil et al. [16] (approximately half vs 33%), a knowledge gap regarding brain death and persistent vegetative state persists. Furthermore, patients often lack an understanding of brain death symptoms and diagnosis. These findings highlight the need for strengthened medical school curricula on brain death to ensure future physicians are well-equipped for accurate diagnosis and effective communication with patients and families.

Student confidence and concerns

Although most students expressed confidence in diagnosing brain death, some harbored anxieties about misdiagnosis and organ procurement while still alive. This highlights the potential psychological impact of incomplete knowledge.

Limitations and future research directions

A limitation of this study is its focus on a single medical school population. A multicenter study would enhance the generalizability of the findings. This research aims to stimulate further investigation into effective educational strategies for improving medical professional and public understanding of brain death.

## Conclusions

This single-center study identified concerning knowledge gaps among fourth-year medical students regarding brain death and organ donation. Students demonstrated limited understanding of key concepts, including legal definitions, diagnostic criteria, and procedures for brain death determination. While some students expressed confidence in diagnosing brain death, anxieties about misdiagnosis and premature organ procurement persisted. These findings suggest a need for revising medical school curricula at this institution to incorporate more comprehensive training on brain death. Future multicenter studies are warranted to determine the generalizability of these findings and to guide the development of effective educational interventions for medical professionals across various institutions.

## Appendices

**Table 4:** The questionnaire

Question number	Question/statement	Response options
1	Gender	Male
		Female
2	Have you completed neurology?	Yes
		No
3	On a scale of 1-5, rate your current understanding of the following:	1 (no knowledge), 2, 3, 4, 5 (expert knowledge)
	* Legal definition of brain death in Saudi Arabia	
	* Presence of a policy on testing and announcing brain death	
	* The prerequisites necessary before conducting a brain death examination	
	* Brainstem reflexes to assess during a brain death examination	
	* The apnea test procedure and its interpretation	
	* Ancillary testing that may be used to support brain death diagnosis	
4	The current diagnostic criterion for brain death is:	Irreversible loss of brainstem function
		Irreversible loss of function of all brain cells
		Irreversible loss of function of the cerebral cortex
		Irreversible loss of function of both hemispheres and the midbrain
		I do not know
5	Do brain-dead patients feel pain?	Yes
		No
		I do not know
6	Can brain-dead patients wake up or regain neurological function?	Yes
		No
		I do not know
7	Which of the following symptoms excludes brain death?	Subtle periodic and rhythmic contractions of facial muscles
		Flexion movements of the fingers of the hand
		Preservation of tendon reflexes
		Present pupillary reaction to light
		All of the above
8	Is it always necessary to perform an instrumental examination in the procedure for pronouncing brain death?	Yes
		No
		Depending on the case
		I do not know
9	Is it possible to maintain pregnancy in women who have been diagnosed with brain death on artificial life support?	No, because the brainstem has died
		Yes, because the brain structures outside the brainstem are responsible for maintaining the pregnancy
		I do not know
10	Do brain death, persistent vegetative state, and locked-in syndrome represent the same medical condition?	Yes
		No
		I do not know
11	Is there a fatwa regarding brain death in Saudi Arabia?	Yes
		No
		I do not know
12	On a scale of 1-5, indicate your level of agreement with the following statements:	1 (strongly disagree), 2, 3, 4, 5 (strongly agree)
	* I am in agreement with brain death diagnosis	
	* I feel confident in my ability to explain the concept of brain death to patients and their families	
	* I believe that organ donation after brain death is an important option for families to consider	
	* Cultural or religious beliefs sometimes make it difficult to discuss brain death with families	
	* I might be misdiagnosed as brain dead and lose the chance of treatment	
	* I might be misdiagnosed as brain dead and my organs and/or tissues might be harvested while I am still alive	
	* My treatment may be terminated prematurely if I declared to donate organs after brain death	
13	Select the primary sources where you have gained knowledge about brain death (more than one answer)	Textbooks
		Faculty staff teaching
		Lectures
		Clinical rotations
		Social medias
		TV shows
		Online medical resources
		Family and friends
